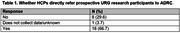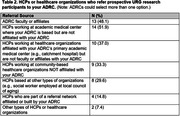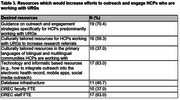# Assessment of Interest and Resources Needed for the Development of Scalable Healthcare Professionals Facilitated Strategies to Diversify Alzheimer’s Disease Research Participation

**DOI:** 10.1002/alz.084479

**Published:** 2025-01-09

**Authors:** Monica W Parker, Crystal M Glover, David K Johnson, Miguel Arce Rentería, Sarah A Biber, Sophia Wang

**Affiliations:** ^1^ Emory University Goizueta Alzheimer’s Disease Research Center, Atlanta, GA USA; ^2^ Emory University, Atlanta, GA USA; ^3^ Rush Alzheimer’s Disease Center, Chicago, IL USA; ^4^ Department of Psychiatry and Behavioral Sciences, Rush University Medical Center, Chicago, IL USA; ^5^ UC Davis Alzheimer’s Disease Center, Walnut Creek, CA USA; ^6^ Columbia University Medical Center, New York, NY USA; ^7^ National Alzheimer’s Coordinating Center, University of Washington, Seattle, WA USA; ^8^ Indiana Alzheimer’s Disease Research Center, Indianapolis, IN USA; ^9^ Indiana University School of Medicine, Indianapolis, IN USA

## Abstract

**Background:**

Increasing underrepresented racial and ethnic minority group (URG) participation in early‐stage Alzheimer’s disease and related dementias (ADRD) research is critical to inclusive characterization of underlying pathology and testing of disease‐modifying treatments. One promising recruitment strategy to accelerate URG participation is for healthcare professionals (HCPs) to facilitate referrals. The use of HCP‐facilitated recruitment strategies across the Alzheimer’s Disease Research Center (ADRC) network, a major referral source for ADRD multisite observational and clinical trials, has not been examined. We hypothesized that there would be interest in the development of scalable HCP‐facilitated recruitment strategies to accelerate URG participation across the ADRC network.

**Methods:**

We emailed Outreach, Recruitment and Engagement (ORE) Cores within the NIA‐funded ADRC network to complete a web‐based REDCap™ survey on their current HCP‐facilitated recruitment strategies for URG participants, resources enhancing use of these strategies, and their interest in strategy development. We conducted descriptive statistics using SPSS 29.0.

**Results:**

Out of 37 ADRCs, 27 (73.0%) completed the survey. Although the majority of ADRCs (66.7%, N = 18) reported HCPs referring URG participants (**Table 1**), they mostly relied on HCP faculty based at the ADRC (48.1%, N = 13) or the ADRC affiliated academic medical center (51.9%, N = 14) (**Table 2**). Nearly all (92.5%, N = 25) ORE Cores expressed interest in participating in or learning more about future efforts to develop HCP‐facilitated recruitment strategies for increasing URG participation. Resources which would increase use of HCP‐facilitated strategies for URG referrals included guidance on outreach and engagement strategies (70.4%, N = 19), culturally tailored resources for HCPs to refer participants (59.3%, N = 16), technology and informatic recruitment strategies (63.0%, N = 17), and staff effort (63.0%, N = 17) (**Table 3**).

**Conclusions:**

Our survey identified key opportunities to develop novel scalable HCP‐facilitated recruitment strategies to accelerate URG participation. Although most ORE Cores expressed interest in expanding their HCP‐facilitated recruitment strategies to have more inclusive research participation, there is need for both higher‐level strategic guidance and ready‐to‐use resources to implement these strategies. Future studies will need to develop and test scalable HCP‐facilitated strategies and resources to systematically accelerate URG research participation.